# Multiliter-Scale Photosensitized Dimerization of Isoprene to Sustainable Aviation Fuel Precursors

**DOI:** 10.1021/acssuschemeng.4c08755

**Published:** 2025-02-04

**Authors:** Leandro Cid Gomes, Sindhujaa Vajravel, William Siljebo, Anup Rana, Tomas Gustafsson, Asimina Bairaktari, Marianne Thomsen, Henrik Ottosson

**Affiliations:** † Department of Chemistry, Ångström Laboratory, Uppsala University, Uppsala 75120, Sweden; ‡ RISE Processum AB, Örnsköldsvik 89122, Sweden; § Department of Food Science, University of Copenhagen, Frederiksberg 1958, Denmark

**Keywords:** cycloalkanes, flow photochemistry, photoreactor design, triplet sensitization, upscaling, monoterpenes

## Abstract

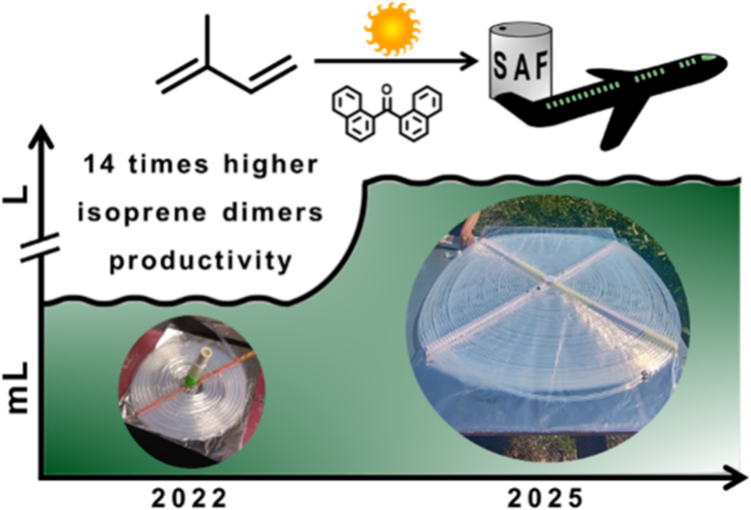

Synthetic routes to sustainable aviation fuels are needed to mitigate the environmental impacts of the aviation sector. Among several emerging methods, the use of light-driven reactions benefits from milder conditions and the possibility of using sunlight to directly irradiate reactants or, alternatively, to power LEDs with a high and constant light intensity. Dinaphthylketone-photosensitized dimerization of isoprene can afford C_10_ cycloalkenes that, after hydrogenation, meet the required properties for jet fuels (strongly resembling Jet-A). Isoprene can be photobiologically produced by metabolically engineered cyanobacteria from the conversion of CO_2_ and water by utilizing solar light, contributing to a carbon-neutral process. The scale-up of such a combined photobiological–photochemical route is essential to bring it closer to the commercial level. Herein, we present the optimization and scale-up of the photosensitized dimerization of isoprene. By designing different reactor setups, flow versus no-flow conditions, and LED lamps (λ_max_ = 365 nm) versus sunlight as the light source, we reached a 2.6 L scale able to produce 61 mL of isoprene dimers per hour, which represents a 14-fold higher productivity compared to our previous results at a smaller scale. We also demonstrated a continuous feed process that converted isoprene into dimers with a 95% yield under LED irradiation. These advancements highlight the potential of light-driven processes to contribute to the energy transition and production of sustainable aviation fuels, making them more viable for commercial use and significantly reducing the environmental impact of the aviation sector.

## Abstract 2

We develop the multiliter-scale photodimerization of isoprene, a critical step of the combined photobiological–photochemical route to sustainable aviation fuels.

## Introduction

The development of sustainable aviation fuels (SAFs) has attracted attention in this century as the carbon footprint of the transport sector needs to be reduced drastically.^1^ The global transport sector was responsible for 22% of the CO_2_ emissions of fuel combustion in 2021, and the aviation sector takes approximately 9% of that share considering jet kerosene combustion, which represents 665 Mt of CO_2_.^2^ The International Air Transport Association (IATA) expects that SAFs will account for 80–90% of the total aviation fuel use by 2050 in order to meet net zero emissions.^3^ Several renewable alternatives have been developed for cars and trucks, such as electrification, hydrogen gas, and liquid fuels, from biobased resources (e.g., methanol and ethanol).^4^ However, these solutions are not straightforward replacements to aviation fuels used for long-haul flights because of the high energy density required and larger battery capacities.^1,5^ In addition, the use of hydrogen gas as a clean fuel still depends on the extensive development of infrastructure and green hydrogen from H_2_O electrolysis driven by windmill energy.^6^ Despite the ongoing development of electric flights and H_2_-powered aircraft,^6,7^ and some hydrogen fuel cell aircrafts predicted to enter the Nordic market by 2045,^8^ significant challenges remain, and SAFs are still more promising as drop-in solutions for long-haul flights where high energy density is crucial.

The current main processes to produce SAFs are hydroprocessing of esters and fatty acids (HEFA), alcohol-to-jet (ATJ), and Fischer–Tropsch (FT) routes.^1^ Vegetable oils such as used cooking oil and soybean oil are the primary feedstocks for HEFA, where they are converted into pure alkanes via metal-catalyzed hydrogenation, decarboxylation, cracking, and isomerization.^9^ In the ATJ route, sugar cane, herbaceous crops, and agricultural waste are used to produce ethanol and isobutanol through fermentation, followed by dehydration and alkene oligomerization to produce long linear alkanes.^5,9^ Agricultural and forest residues along with dedicated crops can be used as lignocellulosic feedstocks in the FT route. In this process, carbon monoxide is produced through pyrolysis and gasification, and then reacted with hydrogen gas to afford liquid hydrocarbons.^10^ A recent study showed that the use of forest residues had a lower environmental impact compared to the other lignocellulosic feedstock.^11^

Although HEFA is currently at commercial level,^5^ it shares similar problems with the other two routes, such as the overinduced land use change and reliance on fossil-based energy and materials inputs.^1^ Despite the environmental impact being lower than that of conventional fossil-based routes, life cycle assessment shows that the impact is highly dependent on feedstock, conversion technology, and geographical aspects,^1,12−14^ making it challenging to further displace CO_2_ emissions. Routes to SAFs that avoid biomass cultivation and processing, such as the use of engineered organisms like cyanobacteria that directly utilize CO_2_ as a feedstock,^15−17^ may offer significant advantages in reducing environmental impacts and displacing CO_2_ emissions.^18,19^

Photosynthetic microorganisms naturally capture CO_2_ and produce organic molecules through photosynthesis using sunlight. For more than a century, photobiological and photochemical routes have been argued to play key roles in the clean energy transition, as first postulated by Ciamician.^20^ Through genetic engineering, the modified strains of photosynthetic organisms can be optimized to produce target molecules, effectively functioning as microbial cell factories.^21^ Cyanobacteria are among the most studied and promising examples of such organisms.^22^ They can be genetically modified to produce diverse and valuable hydrocarbons such as ethanol, butanol, ethylene, isoprene, isobutene, farnesene, and bisabolene, with several recent studies reporting the continuous improvements in the efficiency of these production routes.^23−31^ Advances in metabolic engineering and synthetic biology are driving these enhancements, making cyanobacteria increasingly viable as microbial cell factories.

However, a limitation arises with the size of the hydrocarbon chains that photosynthetic organisms can produce. As hydrocarbon chains grow longer, the cultivation efficiency decreases, as larger chains tend to accumulate within the cells, making extraction more challenging.^32,33^ This accumulation can hinder cell growth and overall productivity, posing challenges for the efficient production of hydrocarbons. Hydrocarbon jet fuel surrogates typically require C_8_–C_15_ chains; therefore, a more suitable approach is to produce smaller volatile hydrocarbons through photosynthesis. These smaller hydrocarbons, such as isoprene (C_5_), can quickly escape from cell cultures,^15,25^ minimizing accumulation issues and facilitating improved cultivation efficiency. These small molecules can then serve as building blocks in subsequent chemical processing steps to afford the desired larger hydrocarbons that are suitable for jet fuel production.

**Figure 1:**
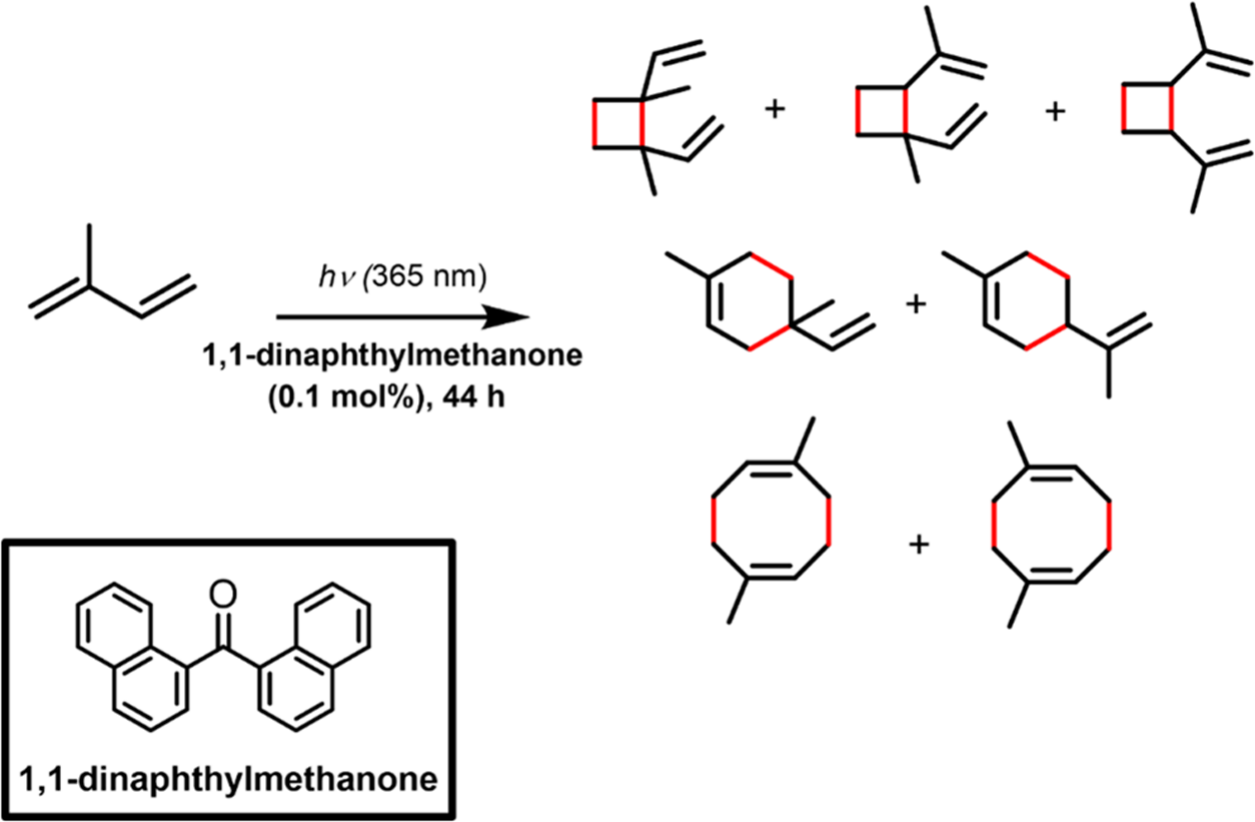
Photosensitized dimerization of isoprene and its products.

Isoprene has been extensively studied as a photobiologically produced feedstock, motivating the development of various methods for its oligomerization.^34−39^ Inspired by earlier works on the photosensitized dimerization of isoprene,^40,41^ our group has developed a combined photobiological–photochemical route to jet fuel.^42^ We demonstrated that the dimerization of isoprene via triplet photosensitization can afford a mixture of dimers which, after hydrogenation, meet and excel the required properties for jet fuels (Figure 1).^42^ In a subsequent study, we demonstrated that, among several small conjugated dienes, isoprene is the optimal choice from both photobiological and photochemical perspectives.^43^ Our solution allows both main steps, the photobiological production of isoprene and its photochemical conversion, to be fully driven by sunlight, providing an opportunity to reduce energy consumption throughout the process. Moreover, the organic photosensitizer used in the dimerization process (i.e., 1,1-dinaphthylmethanone) is reusable and can be synthesized in a straightforward manner contributing to a lower environmental impact. In fact, a life cycle assessment (LCA) of our process showed an 80% reduction in environmental impact compared to fossil-based jet fuel.^42^ However, while this process has been successfully demonstrated on a milliliter scale, significant challenges remain in scaling up the combined photobiological–photochemical route to achieve commercial viability. In this paper, we focus specifically on addressing the photochemical step as a critical component for advancing the process toward large-scale implementation.

A first challenge comes with the development of large-scale photoreactors. Currently, several examples of upscaling photoreactors are found in the literature, in which numbering up and sizing up (length or diameter) strategies are applied.^44,45^ Traditionally, organic photochemical reactions have been performed in batch setups of immersion well reactors or merry-go-around systems with external lamps surrounding the samples.^46^ These systems have a limitation regarding light penetration, which hampers large-scale applications. Falling film reactors have offered a solution for that issue,^47^ although the short residence times require a constant recirculation of the reaction mixture,^46,48^ or the use of high-power light sources.^49^ Booker-Milburn et al. have then developed a new photoreactor by wrapping the light source with a small diameter fluorinated ethylene propylene (FEP) tube,^46^ which benefits from a larger surface area exposed to light, near-UV light transparency and the possibility of continuous flow. However, some problems with the use of FEP tube were later raised by the authors, such as fouling and abrasions, leading to the development of a new photoreactor named Firefly reactor,^50^ in which parallel and continuously connected quartz tubes are used around the light source. Despite the drawbacks, FEP photoreactors are less expensive than the use of quartz tubes and have become very popular, used and adapted by several other authors, enabling kilogram-scale light-driven organic syntheses.^51−54^ More recently, photomicroreactors in different geometries are being developed to promote the industrialization of photochemistry.^55^

Another challenge to scale-up the combined photobiological–photochemical route to jet fuel via isoprene is the volatility of isoprene: while its volatility is beneficial for isoprene extraction from the cell cultures, it can be problematic to handle in the photoreactor under ambient conditions, especially when it requires sunlight exposure conditions in which the reaction temperature can rise up to the boiling point of isoprene (34.07 °C). A third challenge is the direct use of natural sunlight as it is variable in intensity and dependent on local atmospheric conditions.

Addressing all of these challenges is likely to impact the overall costs of the process. In particular, the cost associated with light generation is a critical aspect to consider in light-driven photochemical processes.^56^ In our previous work,^42^ we determined the internal quantum yield of the photosensitized dimerization of isoprene (moles of product produced/mol of photons absorbed) using ferrioxalate actinometry, achieving a value of ϕ = 0.91 when dinaphthylmethanone was employed as the photosensitizer. Although internal quantum yields do not inherently distinguish between photoreactor systems, their impact on the photon cost has been demonstrated in other processes, such as the photoredox-mediated synthesis of ceralasertib, which concluded that light generation costs contribute relatively little to the total costs of a photochemical synthesis compared to other operational costs.^56^

Despite these encouraging results, photon costs could still be significant for certain photoreactions, especially those requiring near-UV light. In our case, we note that commercial near-UV LEDs employed in this study are currently approaching their theoretical conversion efficiency.^56,57^ While this presents limitations in immediate cost reduction, it underscores the importance of exploring alternative strategies to enhance the process’s economic and environmental sustainability.

The sustainability aspects of light-driven processes remain a compelling motivation for further research and development. Leveraging renewable energy sources, such as natural sunlight, has immense potential to reduce both energy costs and environmental impacts. However, the variability of sunlight (in terms of intensity and atmospheric conditions) necessitates the design of more robust and efficient photoreactor systems. This can include the use of less expensive materials for photoreactor manufacturing, such as FEP or alternative polymers, while addressing known issues, such as fouling and abrasion. Additionally, further advancements in photosensitizer development could significantly enhance the process efficiency. These improvements bring the process closer to commercial viability. In this work, we specifically focus on these opportunities and challenges, highlighting pathways for improving the scalability, cost-efficiency, and environmental sustainability of the photobiological–photochemical route to jet fuel via isoprene. Herein we report the multiliter-scale development of the photochemical step in the combined photobiological–photochemical route to jet fuel via isoprene, aiming to advance both the scalability and the sustainability of this pathway as a viable alternative for producing sustainable aviation fuels. We designed a cost-effective and simple multiliter photoreactor that can be easily manufactured and upscaled. The photoreactor can be used for photodimerization under sunlight and LED irradiation with LEDs powered by solar panels. Alongside, we address the practical challenges of working with isoprene in large amounts and under ambient conditions.

## Materials and Methods

### Chemicals and Reagents

To synthesize and purify the photosensitizer 1,1-dinaphthylmethanone, we used, as previously described,^42^ tetrahydrofuran, dichloromethane, dimethylcarbamoyl chloride, 1-bromonaphthalene, *n*-butyllithium, ammonium chloride, dichloromethane, methanol, and ethyl acetate. All of these chemicals and reagent-grade solvents were obtained from Sigma-Aldrich and were used as received. Isoprene (99%, which contains <1000 ppm *p*-*tert*-butylcatechol as a stabilizer) was purchased from Sigma-Aldrich. Prior to use, *p*-*tert*-butylcatechol was removed from isoprene by passing it through activated basic alumina.

### Photoreactors

An RPR-200 Rayonet Photochemical Chamber Reactor was used for the reaction time screening of the photodimerization. The Rayonet photoreactor was equipped with a set of 16 × 24 W UV lamps at 365 nm (purchased from Southern New England Ultraviolet Company). The same reactor was used to test the photodimerization of isoprene under 405 nm light by replacing the set of lamps. The reaction mixture was housed inside a fluorinated ethylene propylene polymer (FEP) tube (O.D. × I.D.: 3.18 mm × 2.1 mm) coiled around a water condenser, with a total volume of the loop size of approximately 20 mL. The distance between the sample solutions and the lamps was 8.5 cm. The same setup with different FEP tubes was used in the investigation of the influence of the FEP tube dimensions. Further photoreactions under LED and sunlight irradiation were performed in a newly designed flat photoreactor of dimensions 1 m × 1 m, with a FEP tube of the O.D. × I.D.: 6.0 mm × 4.0 mm (see the Results and Discussion section). An extrusion 3D printer (Ender 5 Plus, Creality) was used to print the frames to hold the FEP tube. The filament used was polylactic acid (PLA). All CAD designs were made using the online software Tinkercad, and the STL files can be provided by the corresponding author upon request. The flat photoreactor was built using 100 LED lamps with λ_max_ = 365 (LZ1-10UV0R-0000 from Osram Opto Semiconductors, Inc.) and 10 LED drivers (LED50W-072-C0700-D, Thomas Research Products). The LEDs were fixed on aluminum scaffolds (1 m length) using an epoxy adhesive (DP110-GRAY, 3M). A portable spectral radiometer RM12 (350–455 nm) from Opsytec was used to monitor the light intensity of the LED panel and sunlight. A Traceable VWR thermocouple was used to measure the temperature in the reactor under sunlight conditions. A Knauer BlueShadow 10P dual-piston pump was used to establish a flow in the closed-loop and continuous feed setups.

### General Characterization

Isoprene dimers were characterized by gas chromatography–mass spectrometry (GC-MS). The GC-MS system used was provided by an Agilent 7890A GC, equipped with an HP-5 capillary column (30 m × 250 μm × 0.25 μm) and an Agilent 5975 mass selective detector (MSD). Helium was used as the carrier gas. Isolated yields of the dimers were determined gravimetrically.

## Results and Discussion

We first describe the design and construction of the large-scale photoreactor able to accommodate a multiliter-scale photodimerization of isoprene. Then, we demonstrate the use of such a photoreactor under natural sunlight and LED light irradiation. Finally, we address sustainability and economic aspects concerning the upscaling.

### Large-Scale Flat Photoreactor Design

We performed a time screening of the photodimerization of isoprene under 365 nm light in our previous photoreactor (O.D. × I.D.: 3.18 mm × 2.1 mm), and we found that after 44 h of irradiation, the reaction slowed down (Figure S1), so the irradiation time was set to 44 h to compare different FEP tube sizes. A first step to raise the production capacity of the photochemical step is to use tubes with larger dimensions—both their length and the internal diameter. In our previous study,^42^ we have seen a negative effect on the dimerization yield as the dimensions of the FEP tube were increased from O.D. × I.D.: 3.18 mm × 2.1 mm to 6.35 mm × 7.94 mm. In that case, the yield of dimerization dropped from 89 to 48%, while the total reaction volume was increased from 120 to 400 mL. Nevertheless, the actual volume of products was approximately 107 and 192 mL, respectively. So in terms of productivity, there was an improvement despite becoming a less efficient dimerization. We further explored this effect by testing FEP tubes of varying O.D. × I.D. dimensions in a small-scale setup similar to the one reported previously (Table 1).

**Table 1:** Effect of FEP Tubes on the Yield of Isoprene Photodimerization When Used with Different Dimensions

line	brand	I.D. (mm)	wall thickness (mm)	yield (%)	total capacity for 100 m (L)^a^	volume of products (L)^a^
1	Supelco	2.1	0.5	89	0.35	0.31
2	Supelco	3.0	0.4	79	0.70	0.55
3	BOLA	3.0	0.8	57	1.26	0.72
4	BOLA	4.0	1.0	62	1.26	0.78
5	Thermo Scientific	6.4	0.8	48	3.16	1.52

a Calculated theoretical capacities and volume of the products considering a total length of 100 m of the FEP tube. The actual volume for each experiment was lower, as it was limited by the dimensions of the water condenser in which the tube was wrapped. Reactions performed in the RPR-200 Rayonet Photochemical Chamber Reactor (365 nm lamps). Irradiation time was 44 h.

As the diameter of the FEP tubes increased, the dimerization yields decreased, which can be understood by the lower light penetration in the larger tubes. A stronger effect was noticed regarding the wall thickness of the tubes, where a change of 0.4 mm caused a 22% difference in the dimerization yields (Table 1, lines 2 and 3). For all of the tubes tested, we calculated what would be the total reaction volume of a photoreactor built by using 100 m of each tube. Considering the total capacity and yields, we estimated what would be the volume of the products (isoprene dimers) in each of those cases. Surprisingly, though the yields were decreased, in all cases the amount of isoprene dimers produced would be higher, surpassing 1 L for the case of the FEP tube with dimensions of O.D. × I.D.: 6.4 mm × 8.0 mm.

With these results in hand, we moved on to build a multiliter-scale flat photoreactor. We considered a trade-off between moderate yields and total capacity and chose to continue our experiments with the FEP tube with dimensions O.D. × I.D.: 4.0 mm × 6.0 mm. Our choice was also influenced by the commercial availability of the tubes at the time of our experiments design. Nevertheless, as we describe, the custom-made photoreactor that we designed can be easily adapted to tubes of different dimensions.

**Figure 2:**
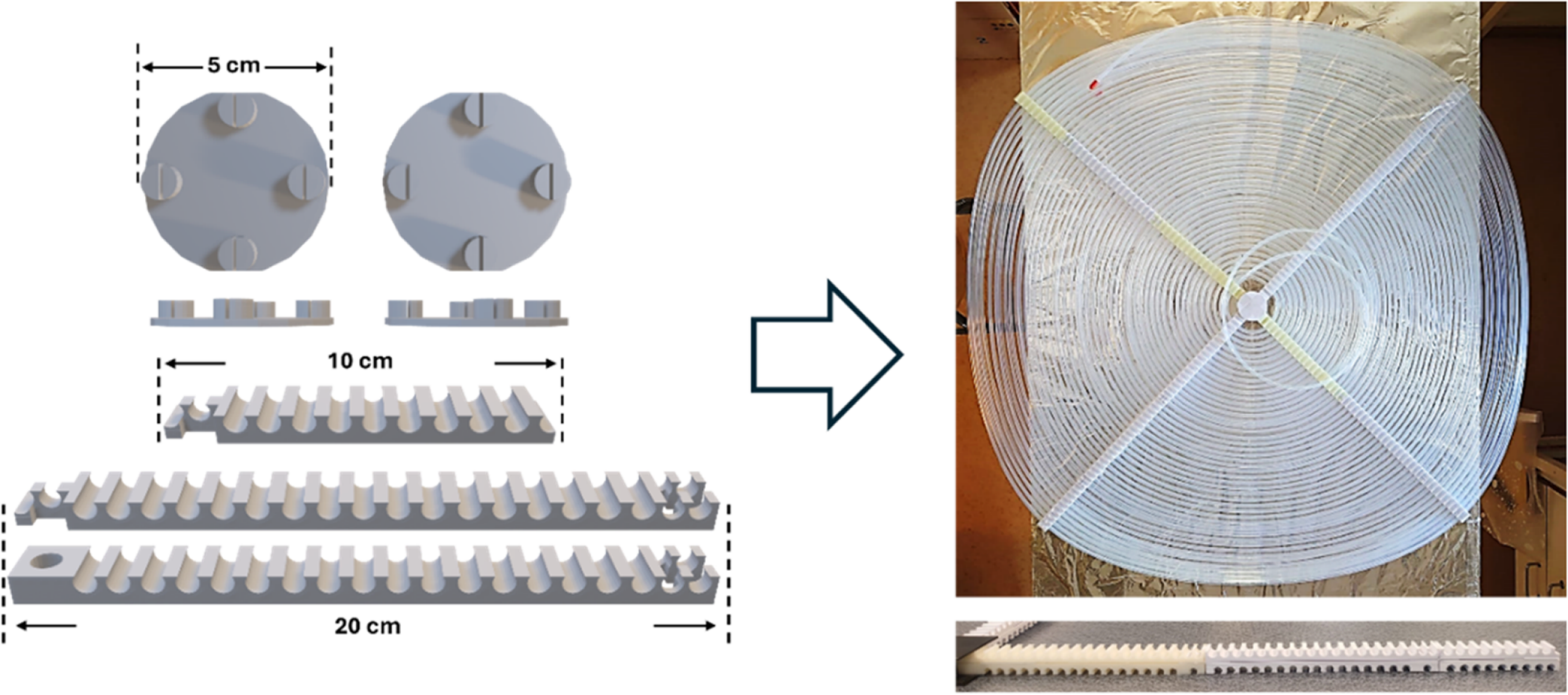
3D images of the modules (left) designed to build the 1 × 1 m^2^ photoreactor frame and a picture of the one-level assembled photoreactor frame with the FEP tube assembled (top right). A picture of the two-level frame is shown on the bottom right.

In order to accommodate 100 m of the FEP tube, we estimated that a new version of our flat photoreactor should have 1 m × 1 m in dimensions. We used the online CAD (computer-aided design) software Tinkercad to design a modular 1 × 1 m support to hold the FEP tubes and printed the modules using an extruding 3D printer with PLA (polylactic acid) filament. Initially, we designed a structure containing pegs to hold the tubes; however, due to the stiffness of the FEP tube and the brittleness of PLA, the support was not successful. We then designed a structure with grooves instead, which was found to hold the tubes properly (Figure 2). The final design was a cross-shaped frame composed of four arms with two segments of 20 cm (inner modules) and one with 10 cm (edge), all of which contained puzzle-like connections to be later assembled. The grooves were also included on the connections to maximize the amount of tube that the support could hold. A two-piece round connector was designed to attach the four arms together.

The custom-made one-level frame had 1.3 L of capacity, which could be doubled to 2.6 L by turning it into a two-level photoreactor. This was made by printing another set of cross-shaped supports and having them attached to the first one (Figure 2, bottom right), creating a double-layer frame. The displacement of the grooves created gaps in between each turn of the FEP tube; therefore, the second level of the frame was printed with mismatched/alternating grooves in relation to the first level, so the gaps from the latter could be more or less aligned to the FEP tubes in the second level. Finally, the photoreactor was placed over a reflective surface (board covered with aluminum foil) in order to enhance the light utilization. Both one- and two-level setups were tested for reactions under natural sunlight and LED irradiation (λ_max_ = 365 nm).

### Natural Sunlight-Driven Reactions

**Figure 3:**
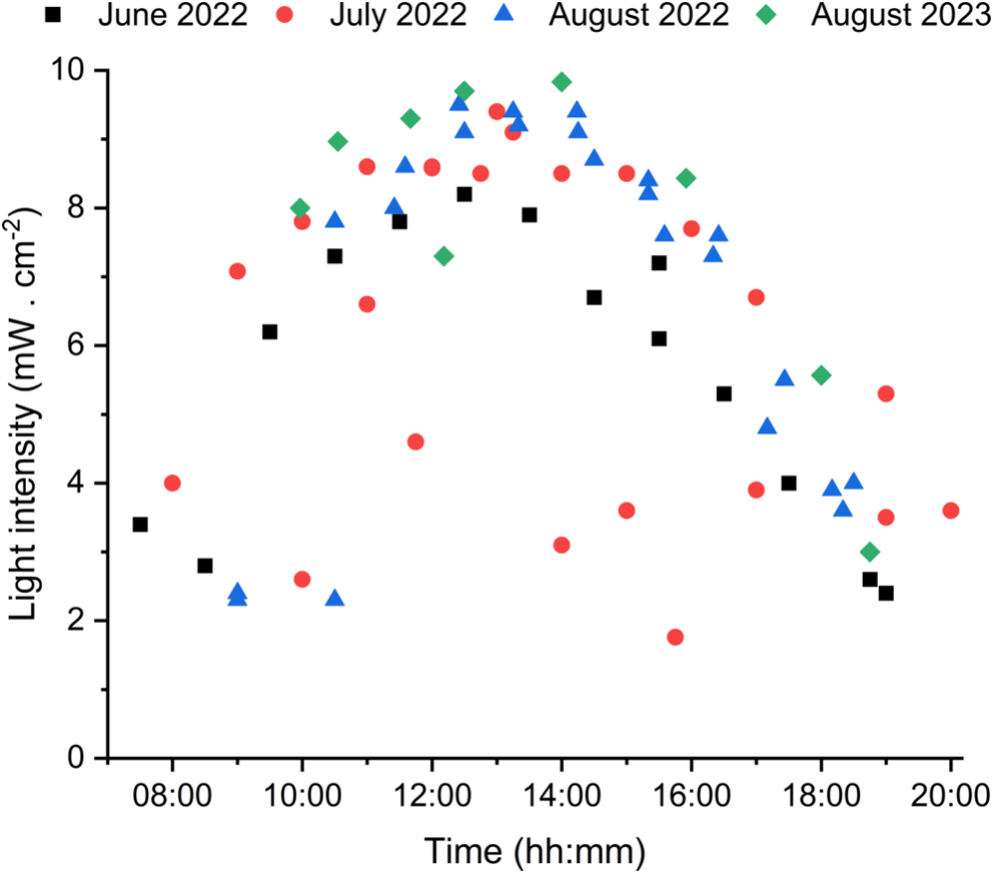
Sunlight intensity over different irradiation periods measured in the range 350–455 nm. Reaction days and total irradiation times: 27th–28th (June 2022, 15 h); 11th–13th (July 2022, 23 h); 23rd–25th (August 2022, 22 h); 17th–20th (August 2023, 30 h).

The flat photoreactor was primarily designed to suit the photosensitized dimerization of isoprene under sunlight. Natural sunlight intensity that reaches the Earth’s surface is expected to vary according to the season, atmospheric conditions, and location. Our experiments were performed at RISE Processum AB in Örnsköldsvik, Sweden, approximately 63°16′16.0″N 18°42′06.3″E. We accounted for the sunlight intensity variation by measuring it in the range between 350 and 455 nm during the irradiation experiments. Typically, the experiments could be performed between 7:00 and 20:30 during the summer seasons of years 2022 and 2023, for a total time of irradiation of 15–30 h for each experiment (over more than 1 day for the same reaction was needed in most cases). The plot of light intensity of the first experiments shows the sunlight intensity varying between 1.8 and 9.5 mW·cm^–2^ (Figure 3). All of the experiments with natural sunlight fell within this range (Figure S2 and Table S1). The reaction temperature throughout the experiments under sunlight irradiation varied from 19 to 36 °C. At temperatures around 25 °C, gas pockets of isoprene were formed during the sample insertion due to isoprene volatility. This issue was initially tackled by cooling the tubes and the isoprene container before inserting the sample. Later, we pressurized the tube at 4 bar using N_2_ gas prior to inserting the sample. This has also aided in faster and more uniform sample filling.

In our first experiment, 1.3 L of a mixture of isoprene and 1,1-dinaphthylmethanone (0.1 mol %) was inserted into the FEP tube of the one-level photoreactor and left exposed to sunlight without any applied flow. After 15 h of irradiation, a yield of 25% was achieved (Table 2, line 1). Next, we repeated the experiment by adding a dual-piston pump to the system to turn it into a closed-loop with a constant flow of 25 mL·min^–1^ (Table 2, line 2). The added flow and a longer irradiation time (23 h) could not improve the yield, which was, in fact, slightly lower (21%) likely due to the light intensity variation. The presence of flow does not largely affect the yield because a laminar flow causes substantial movement only in a narrow region very close to the wall of the tube, i.e., an efficient mass transfer of reactants is not achieved.^58^ For that reason, some small obstacles inside the tube or external clamps to produce kinks could be included to disturb the laminar flow and enhance the mixing through turbulent flow. The mixing quality in photochemical reactors can also be enhanced by a gas–liquid slug flow.^59^ While some losses in photon utilization might be expected due to light scattering, a recent study has demonstrated a counterintuitive result: the introduction of small bubbles in a gas–liquid phase photoreactor can actually enhance photon absorption.^60^ However, the same study also noted that large bubbles can lead to significant photon losses. Applying this approach to our system would require introducing constant inert gas flow, which would increase costs and resource consumption. Additionally, the volatility of isoprene could exacerbate the issue, as unreacted isoprene might escape and reduce its recoverability for reuse in further cycles. On the other hand, when the photochemical step is combined with the photobiological step, the bubbling of isoprene produced by cyanobacteria directly into the reaction mixture may offset these losses and provide an overall improvement in performance. This integration could offer a synergistic solution to maintain the efficient utilization of isoprene while minimizing its escape.

**Table 2:** Performance of Different Photoreactors and Conditions Tested under Sunlight Irradiation^a^

	photoreactor	volume (L)	closed-loop flow (mL·min^–1^)	light intensity (mW·cm^–2^)	time (h)	yield (%)	STY^f^ (mol·h^–1^·m^–3^)
1^b^	one-level	1.3	0	2.4–8.2	15	25	83.3
2^c^	one-level	1.3	25	1.8–9.4	23	21	45.6
3^d^	one-level	1.3	25	2.3–9.5	22	15	34.1
4^d^	clear glass bottle	1.3	stirring	2.3–9.5	22	2.8	6.4
5^d^	clear glass bottle	1.3	without stirring	2.3–9.5	22	2.9	6.6
6^e^	two-level	2.6	50	4.2–10	30	20	33.3

a Reaction temperature: 19–36 °C. Photosensitizer: 1,1-dinaphthylmethanone (0.1 mol %).

b Reaction days: 27th–28th (June 2022).

c Reaction days: 11th–13th (July 2022).

d Reaction days: 23rd–25th (August 2022).

e Reaction days: 17th–20th (August 2023).

f STY = space time yield.

Several alternative reactor designs have also been reported in the literature to improve mixing in laminar flow photoreactors. Examples include coiled-flow inverters,^61^ which promote mixing through repetitive changes in flow direction, spinning disk reactor,^62^ which enhances mass transfer by creating thin films and high-shear flow, and rotating cylinder technology, which improves mixing by rotating the reaction surface itself.^63^ While these reactor types would require larger modifications of our current setup, they represent promising alternatives for enhancing photodimerization efficiency in future work. Careful evaluation of their feasibility and implementation would be necessary to determine the most cost-efficient and scalable solution.

We then performed a third set of experiments with two reactions running in parallel, one in the one-level photoreactor and one with the same volume of sample but inside a clear glass bottle with and without stirring (Table 1, lines 3–5). In this case, although the reaction in the one-level photoreactor showed a lower yield (15%) than the first two experiments, we could observe a clear benefit of having such a setup, since the reaction in the glass bottle had a 5-fold lower yield of isoprene dimers (2.9%). Lastly, we tested the two-level photoreactor under sunlight irradiation with a flow of 50 mL·min^–1^. With a longer reaction time (30 h), the two-level photoreactor reached a yield of isoprene dimers similar to previous experiments in the one-level photoreactor (20%). In this case, the flow was important in order to constantly bring the whole mixture to the upper level, where the exposure to light was higher. For all cases, we also calculated the space time yield (STY), which shows the molar amount of isoprene dimers produced per unit of time and total volume of the reactor. Using this parameter, the same conclusions were reached, except for the case of the two-level reactor (Table 2, line 6), which showed a clear lower STY compared to the one-level cases (Table 2, lines 1–3). Despite that the two-level flat photoreactor showed improvements compared to an ordinary glass bottle, the experiments under natural sunlight irradiation could not reach yields above 25%. Therefore, we continued our studies on the large-scale photodimerization of isoprene using LED lamps as the light source.

### LED-Driven Photodimerization of Isoprene

**Figure 4:**
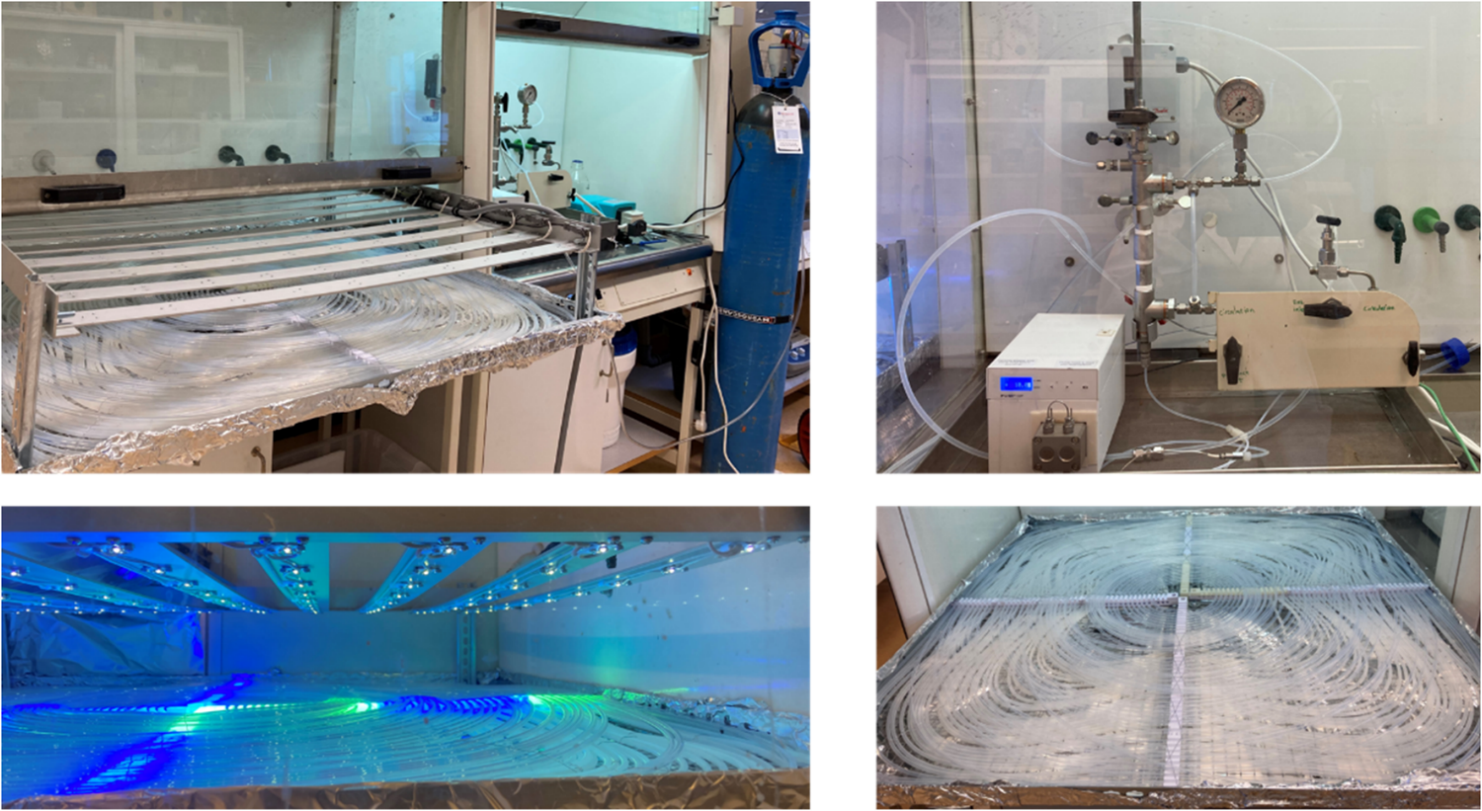
Two-level flat photoreactor system used with the 1 × 1 m^2^ LED panel (left). The dual-piston pump is shown on the top right, and a closer image of the FEP tube assembled on the 1 m × 1 m frame is shown on the bottom right.

We first assessed if LED lamps with longer wavelengths could be suitable for the photodimerization of isoprene. A mixture of isoprene and 1,1-dinaphthylmethanone (0.1 mol %) was inserted in our previous coiled setup and irradiated for 44 h in a Rayonet photoreactor equipped with LED lamps of λ_max_ = 405 nm. The yield of isoprene dimers was 53%, therefore almost half of the one found in the same conditions for irradiation under λ_max_ = 365 nm. Although this result is improved compared to the sunlight experiments, the 365 nm wavelength was still the optimal choice to match the absorbance range of 1,1-dinaphthylmethanone (Figure S3). Therefore, we built a 1 m × 1 m panel containing 100 (10 × 10) equally spaced LED lamps (λ_max_ = 365 nm) and tested the photodimerization of isoprene in the two-level photoreactor (Figure 4). The upper level of the FEP tube was placed at a distance of 14 cm below the LED panel, and the experiments were performed at room temperature in the lab.

In the first experiment, the light intensity was set to 9 mW·cm^–2^ and the flow (closed-loop) was set to 50 mL·min^–1^. After 24 h of irradiation, a yield of 47% isoprene dimers was reached (Table 3, line 1). Repeating this experiment with a higher light intensity (15 mW·cm^–1^) increased the yield to 70%, while extending the time to 38 h provided 90% yield of isoprene dimers (Table 3, lines 2 and 3, respectively). We managed to produce 2.3 L of isoprene dimers in 38 h, reaching our initial goal of upscaling the photochemical step of the combined photobiological–photochemical route to a multiliter scale. Once again, we calculated the space time yields for each condition tested. We found that extending the residence time from 24 to 38 h gives a lower STY, which shows that the shorter time would be preferred in terms of isoprene dimers produced per hour and volume of reactor. On the other hand, because the costs of light generation have a small impact on the overall costs,^56^ the extended reaction time (38 h) might still be an acceptable case, where the cost for removing unreacted isoprene from the dimers mixture would be lower than for the case of shorter time (24 h) due to the lower conversion yield.

**Table 3:** Performance of the Two-Level Photoreactor under Different Light Intensities of 365 nm Light (LED) and Varying Flow Conditions^a^

	photoreactor	volume (L)	flow (mL·min^–1^)	light intensity (mW·cm^–2^)	time (h)	yield (%)	STY^d^ (mol·h^–1^·m^–3^)
1	two-level	2.6	50^b^	9	24	47	97.9
2	two-level	2.6	50^b^	15	24	70	145.8
3	two-level	2.6	50^b^	15	38	90	118.4
4	two-level	5.0	5^c^	9	16	25	
5	two-level	5.0	1^c^	9	53	95	

a Room temperature. Photosensitizer: 1,1-dinaphthylmethanone (0.1 mol %)

b Closed-loop.

c Continuous feed.

d STY = space time yield.

Finally, we tested a continuous feed setup, which could be ideal for combination with the photobiological preceding step. In this case, a reservoir containing 5 L of the isoprene and 1,1-dinaphthylmethanone (0.1 mol %) mixture was connected to the pump, and the feeding flow was set to 5 mL·min^–1^. The sample entered the reactor from its center, and then we turned on the LED panel (light intensity of 9 mW·cm^–1^) as soon as the sample started flowing into the FEP tube. After 16 h of irradiation, 100 mL had come out of the photoreactor. We collected this volume of reacted mixture and checked the isoprene dimer content, in which we found a low yield of 25% (Table 3, line 4). When decreasing the feeding flow to 1 mL·min^–1^, the same procedure took 53 h with a much higher yield of isoprene dimers (95%).

In all our photoreactions, the unreacted isoprene could be easily distilled off, separating it from the dimers and enabling refeeding to a next batch of reactions. Likewise, the photosensitizer can be recovered from the isoprene dimer mixture by further distillation of the dimers or by column chromatography, providing a way to reuse the photosensitizer in several reactions.

### Sustainability and Scalability Considerations

An evaluation of the scalability and sustainability of alternative processes to SAFs, such as the combined photobiological–photochemical route proposed here, must consider the complex synergies and trade-offs between energy and the Sustainable Development Goals (SDG).^64^ This requires integrating anthropogenic, environmental, societal, and economic factors into the assessment.^65^

A critical consideration for scaling the process involves the choice between scale-up (increasing the size of photoreactors) and scale-out (replicating smaller, modular systems). Scale-out approaches may offer advantages such as improved redundancy, easier local deployment, and enhanced adaptability to region-specific resources. For instance, systems could be tailored to utilize CO_2_ from local industrial emissions and harness abundant natural sunlight, particularly in regions of the Global South where solar energy is a significant resource (SDGs 7 and 10).

Future studies should include the determination of external quantum yield (moles of product produced/mol of incident photons), a critical metric for assessing photon costs and the economic feasibility of the process.^56^ Enhancing the cost-efficiency of the process fully powered by natural sunlight could involve innovative light-capture strategies, such as holographic reflectors, luminescent solar concentrators, and solar-tracking setups.^66^ The integration of advanced light management techniques with scalable reactor designs will be essential to ensure consistent photon utilization under varying environmental conditions, addressing the goals of industrial innovation and infrastructure (SDG 9).

Our process has several potential sustainability advantages compared to conventional SAF pathways: (i) avoided extensive land use by eliminating the need for biomass cultivation and processing;^67,68^ (ii) reduction of the risk of novel entities that could lead to even more severe transgression of the planetary boundaries, such as chemical pollutants and ecosystem disruptions (SDG 3, 14, and 15);^69^ (iii) elimination of environmentally costly input resources, such as inert gases and noble metal catalysts often required in alternative routes (SDG 12). These factors position our process as a promising alternative that aligns with circular resource management and localized production, provided that the rebound effect of a final SAF produced is negligible.^70−72^ Furthermore, the enhanced photosensitized dimerization offers versatility beyond jet fuel. Dimers of larger biobased molecules, such as mono- and sesquiterpenes, can be produced and, after hydrogenation, utilized as high-energy-density fuels and lubricant oils, broadening the impact across multiple sectors.^73^

While our previous LCA for the photobiological–photochemical process showed promising results,^42^ it was based on small-scale data. Therefore, the present analysis is qualitative, and future quantitative sustainability assessments as well as cost evaluation must be performed.^36^ The multiliter-scale advancements reported here will strengthen future prospective Life Cycle Assessments (LCA) and economic viability assessments by improving the Life Cycle Inventory (LCI) data model of both the photochemical and photobiological steps at scale to provide actionable insights for advancing this process toward commercial readiness.

Nevertheless, certain macrolevel challenges persist. For example, while LEDs are energy-efficient, their integration into the photobiological–photochemical process relies on clean energy inputs. This, in turn, depends on the development and accessibility of renewable energy infrastructure (e.g., from solar, wind, or hydropower plants) that avoids disruption of local ecosystems and arable land (SDG 7). The interdependencies between renewable energy production and other SDGs highlight the need for a broader systems perspective. For instance: Efficiency improvements, which may inadvertently compromise sustainability goals, such as biodiversity or social equity, and implementation of economic incentives and policy frameworks, which are designed to prioritize sustainability over purely economic optimization.

While isoprene dimerization can also be accomplished thermally (e.g., heating at 200 °C for 1.5 h) or through metal- or acid-catalyzed processes,^34−39,74^ these methods should not be viewed as competitive processes, but rather as complementary to solar-driven approaches. The most advantageous process will depend on local conditions, including energy availability, cost, and environmental considerations, which underscores the importance of partnerships between innovative technologies and industries (SDGs 9 and 17). By adopting a broad sustainability perspective that integrates technological, economic, social, and environmental dimensions, industries can collaborate to identify the most impactful pathways for SAF production. Such an approach ensures that the benefits of this process are maximized across multiple SDGs.

## Conclusions

Our combined photobiological–photochemical route to jet fuel via isoprene holds good potential as a replacement for current fossil jet fuels.^42^ In this work, we successfully developed a multiliter-scale photoreactor for the photochemical step capable of operating under both natural sunlight and LED irradiation. We achieved a 14-fold increase in the number of isoprene dimers produced per batch. Based on our findings with larger FEP tubes, further improvements are likely achievable by employing tubes with larger internal diameters (I.D.) and thinner walls as well as introducing methods to disturb laminar flows and enhance mass transport (mixing). An additional advantage found in our best-performing setup is its potential to couple seamlessly with a photobioreactor, enabling a continuous feed of reactants and efficient recovery of products.

The present results also reaffirm that the photosensitized dimerization of isoprene can be conducted under natural sunlight. However, the current photosensitizer (1,1-dinaphthylmethanone) has an absorption range that limits the full utilization of the solar spectrum. Moreover, the variable sunlight intensities in Sweden pose a challenge for consistent operation. Addressing these limitations will require further development of triplet sensitizers with broader absorption capabilities, enabling the harvest of a wider portion of the solar spectrum, increasing photon utilization, and reducing energy demands.

Nonetheless, the use of LED lamps as light sources presents a viable alternative. By coupling LED systems with solar panels to power them, this approach can indirectly harness sunlight, significantly contributing to the sustainability of the process.

## Supporting Information Available

Additional synthetic procedure details; sunlight intensity measured during photodimerization; time screening of isoprene photodimerization; UV–vis spectrum of 1,1-dinaphthylmethanone; picture of the experimental setup of photodimerization under natural sunlight; and GC-Ms data. (PDF)
