# Supplementary material for: Multiliter-Scale Photosensitized Dimerization of Isoprene to Sustainable Aviation Fuel Precursors
Source: ACS Sustain Chem Eng. 2025 Feb 4;13(6):2467–76. doi: 10.1021/acssuschemeng.4c08755 (PMC11837299; doi:10.1021/acssuschemeng.4c08755)
Supplement: Supplementary file 1 — sc4c08755_si_001.pdf [file sc4c08755_si_001.pdf]

Supporting Information

Multiliter Scale Photosensitized Dimerization of Isoprene to Sustainable Aviation Fuel Precursors

Leandro Cid Gomes,[a‡] Sindhujaa Vajravel,[a‡] William Siljebo,[b] Anup Rana,[a]
Tomas Gustafsson,[b] Asimina Bairaktari,[c] Marianne Thomsen[c] and Henrik Ottosson*[a]

[a] Department of Chemistry – Ångström Laboratory, Uppsala University, Box 523, 751 20 Uppsala, Sweden. [b] RISE Processum AB, Box 70, 891 22 Örnsköldsvik, Sweden.
[c] Department of Food Science, University of Copenhagen, 1958 Frederiksberg, Denmark
E-mail: henrik.ottosson@kemi.uu.se

Number of pages: 7

Number of figures: 5

Number of tables: 1

Table of contents

General synthetic procedure for isoprene dimerization..S3

Table S1: Sunlight intensity measured in the range 350 – 455 nm during photoreactions.....S4

Figure S1: Isoprene conversion into dimers over irradiation time. Photosensitizer: 1,1-dinaphthylmethanone (0.1 mol%), light source at 365 nm and using the Rayonet photoreactor. S5

Figure S2: Sunlight intensity measured in the range 350 – 455 nm during photoreactions (see Table S1)..S5

Figure S3: UV-Vis absorption spectrum of 1,1-dinaphthylmethanone in dichloromethane. .S6

Figure S4: The chromatograms and mass spectra showing isoprene dimers produced via photosensitized dimerization of isoprene by 1,1-dinaphthylmethanone (0.1 mol%) in different reactors and light sources: Rayonet reactor (365 nm, control), LED panel and sunlight. The retention times of the dimers are from 2.95 to 4.28 minutes. The dimers were formed under all conditions with the same distribution. A-B: Rayonett reactor. C-D: Sunlight. E-F: LED panel (9 mW/cm^2). G-H: LED panel (15 mW/cm^2). S7

Figure S5: Typical setup used in the photodimerization of isoprene under natural sunlight irradiation. On the right side of the figure, it is shown the solar panel used to power the double piston pump needed to establish the flow...S7

General synthetic procedure for isoprene dimerization

In a typical experiment, a non-degassed isoprene sample was first passed through a pack of activated basic alumina to remove the stabilizer *p*-tert-butylcatechol. Then the stabilizer-free isoprene was mixed with the photosensitizer 1,1-dinaphthylmethanone to reach a concentration of 0.1 mol%. When the photosensitizer was completely dissolved, the mixture was transferred into the photoreactor. At the end of the reaction, the unreacted isoprene was removed under reduced pressure.

Table S1: Sunlight intensity measured in the range 350 – 455 nm during photoreactions.

Date (dd-mm-aaaa)	Time (hh:mm)	Light intensity (mW . cm^{-2})	Date (dd-mm-aaaa)	Time (hh:mm)	Light intensity (mW . cm^{-2})
**June 2022**			**August 2022**		
27-06-22	15:30	7.2	23-08-22	14:14	9.4
27-06-22	17:30	4.0	23-08-22	15:20	8.4
28-06-22	7:30	3.4	23-08-22	16:20	7.3
28-06-22	8:30	2.8	23-08-22	18:10	3.9
28-06-22	9:30	6.2	24-08-22	9:00	2.4
28-06-22	10:30	7.3	24-08-22	10:30	2.3
28-06-22	11:30	7.8	24-08-22	11:25	8.0
28-06-22	12:30	8.2	24-08-22	12:25	9.5
28-06-22	13:30	7.9	24-08-22	13:15	9.4
28-06-22	14:30	6.7	24-08-22	14:15	9.1
28-06-22	15:30	6.1	24-08-22	15:20	8.2
28-06-22	16:30	5.3	24-08-22	16:25	7.6
28-06-22	18:45	2.6	24-08-22	17:26	5.5
28-06-22	19:00	2.4	24-08-22	18:30	4.0
**July 2022**			25-08-22	9:00	2.3
11-07-22	11:45	4.6	25-08-22	10:30	7.8
11-07-22	15:00	3.6	25-08-22	11:35	8.6
11-07-22	15:45	1.8	25-08-22	12:30	9.1
11-07-22	17:00	3.9	25-08-22	13:20	9.2
11-07-22	19:00	3.5	25-08-22	14:30	8.7
11-07-22	20:00	3.6	25-08-22	15:35	7.6
12-07-22	8:00	4.0	25-08-22	17:10	4.8
12-07-22	9:00	7.1	25-08-22	18:20	3.6
12-07-22	10:00	7.8	**August 2023**		
12-07-22	11:00	8.6	17-08-23	18:45	3.0
12-07-22	12:00	8.6	18-08-23	9:58	8.0
12-07-22	12:45	8.5	18-08-23	10:33	9.0
12-07-22	13:15	9.1	18-08-23	12:30	9.7
12-07-22	14:00	8.5	18-08-23	14:00	9.8
12-07-22	15:00	8.5	18-08-23	15:55	8.4
12-07-22	16:00	7.7	18-08-23	18:00	5.6
12-07-22	17:00	6.7	19-08-23	11:40	9.3
12-07-22	19:00	5.3	20-08-23	12:11	7.3
13-07-22	10:00	2.6			
13-07-22	11:00	6.6			
13-07-22	12:00	8.6			
13-07-22	13:00	9.4			
13-07-22	14:00	3.1			

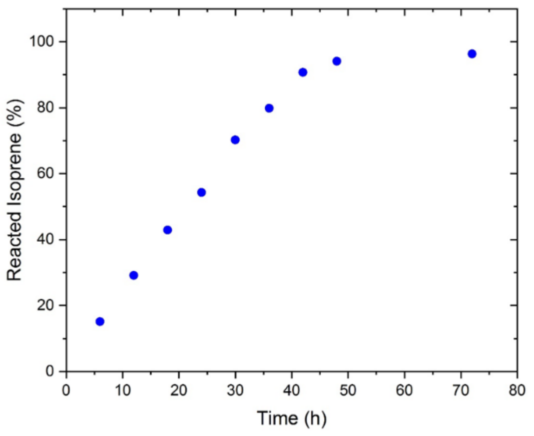

Figure S1: Isoprene conversion into dimers over irradiation time. Photosensitizer: 1,1-dinaphthylmethanone (0.1 mol%), light source at 365 nm and using the Rayonet photoreactor.

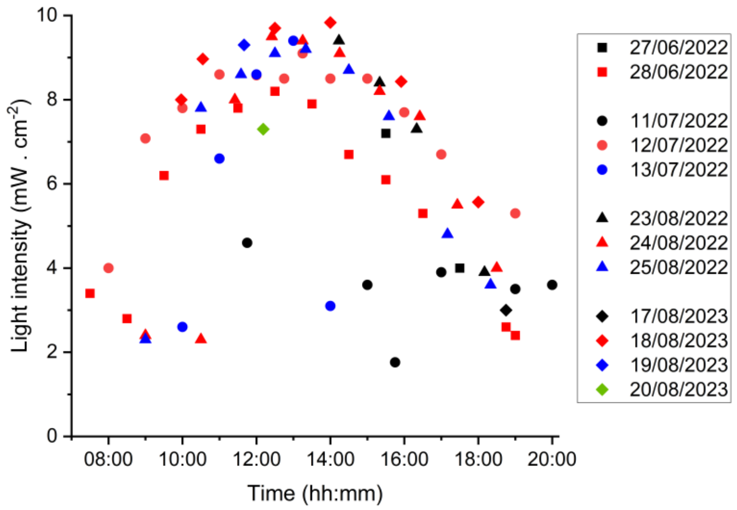

Figure S2: Sunlight intensity measured in the range 350 – 455 nm during photoreactions (see Table S1).

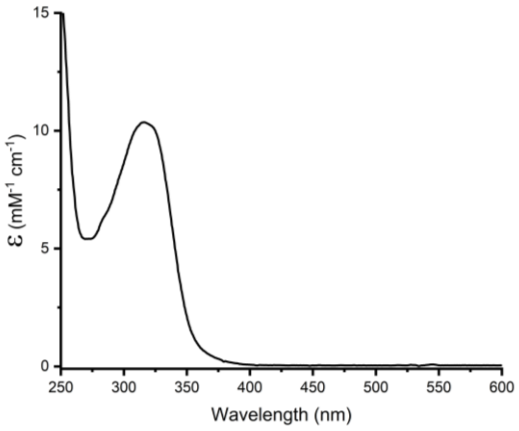

Figure S3: UV-Vis absorption spectrum of 1,1-dinaphthylmethanone in dichloromethane.

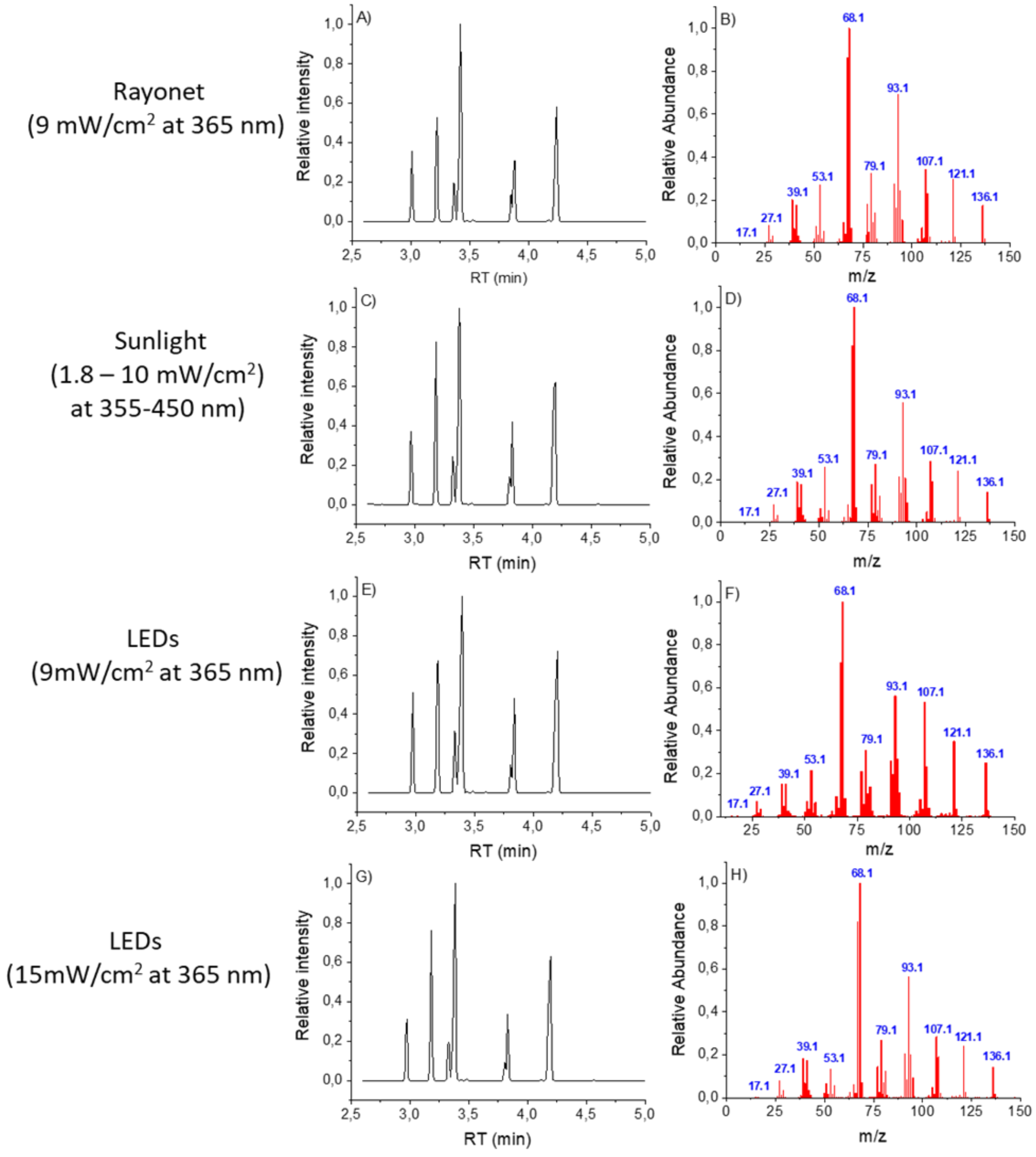

Figure S4: The chromatograms and mass spectra showing isoprene dimers produced via photosensitized dimerization of isoprene by 1,1-dinaphthylmethanone (0.1 mol%) in different reactors and light sources: Rayonet reactor (365 nm, control), LED panel and sunlight. The retention times of the dimers are from 2.95 to 4.28 minutes. The dimers were formed under all conditions with the same distribution. A-B: Rayonett reactor. C-D: Sunlight. E-F: LED panel (9 mW/cm²). G-H: LED panel (15 mW/cm²).

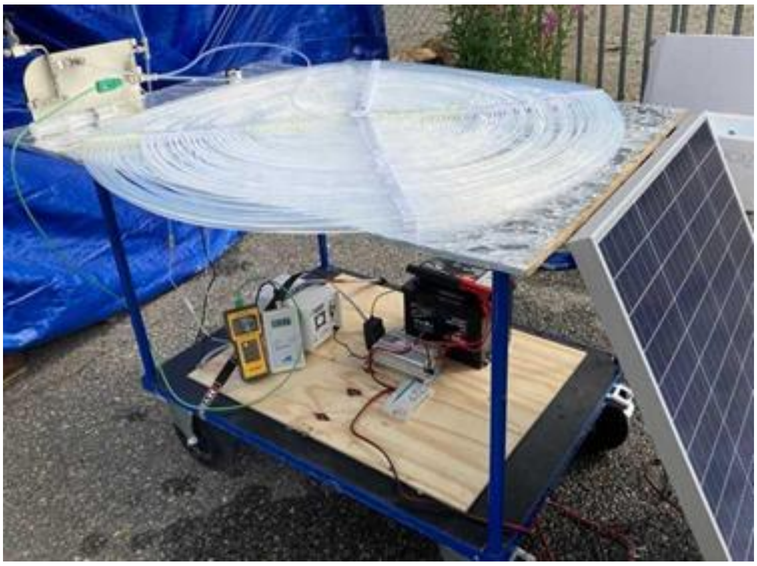

Figure S5: Typical setup used in the photodimerization of isoprene under natural sunlight irradiation. On the right side of the figure, it is shown the solar panel used to power the double piston pump needed to establish the flow.
